# TREM2 improves microglia function and synaptic development in autism spectrum disorders by regulating P38 MAPK signaling pathway

**DOI:** 10.1186/s13041-024-01081-x

**Published:** 2024-02-26

**Authors:** Yi Tian, Xiao Xiao, Weiliang Liu, Shanqing Cheng, Na Qian, Ling Wang, Yang Liu, Rong Ai, Xiaoping Zhu

**Affiliations:** 1 School of Pediatrics, Guizhou Medical University, Guiyang City, China; 2 Department of Pediatrics, The Affiliated Hospital of Guizhou Medical University, No. 28 Guiyi Street, Yunyan District, 550004 Guiyang City, China

**Keywords:** Autism spectrum disorder, Triggering receptor expressed on myeloid cells-2, Microglial cells, Synaptic, Valproic acid, P38 MAPK pathway

## Abstract

**Background:**

Autism spectrum disorder (ASD) encompasses a diverse range of neurodevelopmental disorders, but the precise underlying pathogenesis remains elusive. This study aim to explore the potential mechanism of TREM2 in regulating microglia function in ASD.

**Materials and methods:**

The offspring rat model of ASD was established through prenatal exposure to valproic acid (VPA), and the behavioral symptoms of the ASD model were observed. On postnatal day (PND) 7 and PND 28, the effects of prenatally exposure to VPA on synaptic development and microglia phenotype of offspring rats were observed. Primary microglia were cultured in vitro. Lentivirus and adenovirus were utilized to interfere with TREM2 and overexpress TREM2.

**Results:**

Prenatally VPA exposure induced offspring rats to show typical ASD core symptoms, which led to abnormal expression of synapse-related proteins in the prefrontal cortex of offspring rats, changed the phenotype of microglia in offspring rats, promoted the polarization of microglia to pro-inflammatory type, and increased inflammatory response. The experimental results in vitro showed that overexpression of TREM2 could increase the expression of Gephyrin, decrease the content of CD86 protein and increase the content of CD206 protein. In addition, after the expression of TREM2 was interfered, the content of p-P38 MAPK protein increased and the content of p-ELK-1 protein decreased.

**Conclusion:**

The protective influence of TREM2 on the VPA-induced ASD model is attributed to its inhibition of the P38 MAPK pathway, this protective effect may be achieved by promoting the polarization of microglia to anti-inflammatory phenotype and improving the neuronal synaptic development.

## Introduction

Autism spectrum disorder (ASD) encompasses a diverse range of neurodevelopmental conditions, characterized primarily by varying levels of linguistic and social impediments, repetitive stereotypical behaviors, and limited areas of interest [1]. Currently, the etiology and pathogenesis of ASD are believed to arise from the interplay of genetic and environmental factors during early developmental stages; however, the precise mechanisms underlying its pathogenesis remain elusive [2].

Microglia, constituting 10-15% of all cells in the adult brain, serve as innate immune effector cells within the central nervous system. In addition to their primary role in defending against central nervous system damage, microglia also actively engage in synapse formation and elimination, developmental pruning, and neurogenesis [3]. After activation, microglia can protect brain function by releasing cytokines, chemokines and growth factors. Studies have shown that compared with non-ASD patients, the density of microglia in two different cortical regions (frontal lobe and visual cortex) of ASD patients is significantly increased [4]. Another study found that ASD mice with TMEM59 knockout in microglia showed enhanced excitatory synaptic transmission, increased density of dendritic spines and impaired synaptic phagocytosis [5].

Triggering receptor expressed on myeloid cells-2 (TREM2), a member of the immunoglobulin superfamily receptors, which exists in various myeloid cells including microglia, osteoclasts and macrophages [6]. TREM2 inhibits the secretion of inflammatory mediators by binding to DNAX-activating protein of 12 kDa (DAP12), which plays a very important role in inflammatory response and natural immune response [7]. In addition, TREM2 has phagocytosis, and its mutation has been identified as a risk factor for many neurodegenerative diseases [8]. According to many reports, TREM2 has the potential to modulate microglial activation, inflammatory response, phagocytosis, myelin sheath re-formation, and axon integrity, thereby contributing to enhanced neural functioning [9–10]. Therefore, TREM2 has become a hot target in the field of nervous system in recent years.

Filipello F et al. reported that the expression of TREM2 protein was decreased in ASD patients, which was negatively correlated with the severity of ASD symptoms, and the lack of TREM2 led to the decrease of microglia activation during early brain development [11]. Luo L et al. proved for the first time that prenatal exposure to Valproic acid (VPA) may affect the activation, polarization and synaptic pruning of microglia through down-regulation of TREM2, resulting in ASD symptoms in rat offspring [12]. In this study, the previous studies were confirmed, and the offspring ASD rat model was established by prenatal exposure to VPA, and the ASD-related behavior of the offspring rats was observed, and TREM2 was interfered and overexpressed to observe the regulatory role of TREM2 in microglia function. On the basis of previous studies, this study deepened the understanding of the mechanism of TREM2 in ASD, and discussed the role of TREM2 in ASD through the P38 MAPK signaling pathway.

## Methods

### Animals

12 healthy Wistar rats aged 8–10 weeks were housed overnight in cages with a male to female ratio of 2:1. On the following day, the male rats were removed, and the vaginal suppository of the female rats was examined in separate cages. The successful attachment of the vaginal suppository was considered indicative of a successful pregnancy. Rats in the 12.5-day gestation period were randomly assigned to two groups: Model group, in which intraperitoneal injections of VPA (600 mg/kg, 50 mg/ml) were administered, the offspring designated as the VPA group; Control group, in which the same dose of sterile saline was injected intraperitoneally, the offspring designated as the Con group. The offspring were weaned at postnatal day 21 (PND 21). For this study, only male offspring rats were chosen, 5 offspring rats in each group. The behavioral symptoms associated with the ASD model were then observed at PND 35. Prior to the experiment, a period of 7 days was allocated for the adaptive rearing of all rats. The rats were provided with standard feed in a sanitized environment and were granted unrestricted access to food and water. This study was approved by the Management and Use Committee of Experimental Animals of Guizhou Medical University (NO.2,100,584).

### Behavioral test

(1) Three-chamber test: The experimental box was partitioned into chamber A, central chamber and chamber B. In the social ability tracking test stage, the time that the rats stayed in the central chamber and the time of olfactory contact communication with stranger 1 were observed. In the social novelty test stage, the olfactory contact communication time between the tested mice and stranger 1 or stranger 2 were observed. (2) Elevated plus maze test: The rats were positioned at the central of the maze, facing an open arm. The retention time in the open arm of rats was recorded. (3) Open field test: The rats were situated within the central area and granted unrestricted movement for 10 min. The retention time of the rats in the central area was recorded. (4) Marble-burying test: A cushion was placed inside the cage, upon which glass beads were deposited. Total number of marbles buried more than 2/3 within 30 min was recorded. (5) Self-grooming test: The rats were transferred to an empty box and granted unrestricted movement for 10 min The cumulative time of self-grooming was recorded within 10 min.

### Tissue sample collection

On PND 7 and PND 28, 6 male offspring rats were anesthetized and executed. The prefrontal cortex (PFC) tissue of the rats was preserved on ice, frozen in liquid nitrogen and stored at -80℃. The offspring rats were randomly divided into 4 groups, 3 rats in each group. (1) normal PND 7 group; (2) normal PND 28 group; 3.ASD model PND 7 group; 4.ASD model PND 28 group.

### ELISA assay

The levels of Interleukin-1β (IL-1β) and IL-4 in brain tissue of PFC area region were measured. IL-4 ELISA kit (MM-0191R2) and IL-1β ELISA kit (MM-0047R2) (Jiangsu Enzyme Immunity Industry Co., Ltd.). Frozen brain tissue was taken, homogenized, and centrifuged to obtain the supernatant. Each standard and sample well was then treated with 100 µl of horseradish peroxidase labeled detection antibody and incubated for 60 min. The liquid was discarded, and the board was washed. Substrates A and B (50 µl) were added to each well and incubated. 50 µl of stop solution was added to each well, and the OD value at a wavelength of 450 nm was determined.

### Transmission electron microscope observation

The PFC tissue of rat brain was trimmed and sectioned into ultrathin slices. The tissue was subjected to double staining using uranium acetate and lead citrate, and the resulting ultrastructure was examined using a transmission electron microscope.

### Immunofluorescence

Immunofluorescence double staining was used to observe the protein expression of brain PFC microglia phenotype IBA-1 + CD86, IBA-1 + CD206, IBA-1 + TREM2, TREM2 + DAP12. Brain tissue was isolated, fixed and dehydrated, and 8 μm sections were prepared. A sealing solution containing 5% BSA and 0.2% TritonX-100 was added. The primary antibody IBA-1, CD86, CD206, TREM2 and DAP12 were added separately and incubated. A mixed solution of secondary antibodies was added and incubated. The tablets were sealed with DAPI.

### Cell culture and grouping

Both normal newborn rats and ASD newborn rats were euthanized, and the prefrontal cortex (HPC) was isolated. The brain tissue was sectioned, added with 0.25% trypsin, and homogenized through repeated agitation. Once the cells prone to adhesion had proliferated, the cell suspension containing non-adherent neurons and microglia was transferred to a separate cell culture vessel, and the mixed glial cells adhered to the surface during cultivation. The unattached cell suspension containing neurons was transferred to a cell culture bottle that had been pretreated with polylysine. Once the majority of neurons adhered to the wall, the culture medium was changed to a solution containing B27 to facilitate continued culture, with the majority of cells being neurons. The mixed glial cells that adhered to the wall after 4 h were cultured, and microglia were obtained using a shaking method.

The experiments were categorized into different groups: control microglia + control neurons (Con), ASD microglia + ASD neurons (ASD), ASD microglia + TREM2 overexpressed no load + ASD neurons (ASD + Trem2-NC), ASD microglia + TREM2 overexpressed adenovirus + ASD neurons (ASD + Trem2-OE) and ASD microglia + TREM2 interference vector + ASD neuron (ASD + Trem2-siRNA).

### TREM2 interference and over-expression technology processing

With TREM2 as the target gene (NM_001106884.1, Gene ID: 301,227), the interference sequence was designed and synthesized by Anhui General Company. During usage, the dry powder siRNA underwent centrifugation at a speed of 3000 rPm for 1 min. ddH2O was added in accordance with the instructions, and the resulting mixture was agitated for 10 min to ensure complete dissolution of all dry powder adhering to the inner surface of the tube. A 20 μm siRNA solution was prepared, which was subjected to subpackaging and stored at -20 °C. The cells were transfected with the siRNA solution, and RNA were collected 48 h post-transfection.

The interference agent sequence employed was as follows: NC: Forward (5’-3’):UUCUCCGAACGUGUCACGUTT, Reverse (5’-3’):ACGUGACACGUUCGGAGAATT; TREM2(Norway rat) siRNA-603: Forward (5’-3’):CCGAGGAGUCAGAGAGUUUTT, Reverse (5’-3’):AAACUCUCUGACUCCUCGGTT; TREM2(Norway rat) siRNA-234: Forward (5’-3’):CCACAGUGCUGCAGGGUGUTT, Reverse (5’-3’):ACACCCUGCAGCACUGUGGTT; TREM2(Norway rat) siRNA-404: Forward (5’-3’):CAGAAUGGGAGCACGGUCATT, Reverse (5’-3’):UGACCGUGCUCCCAUUCUGTT.

The cells were transfected with adeno-associated virus encoding TREM2 (GeneChem Biotech, China) or adenovirus empty vector for 48 h. The titer of overexpressed TREM2 adenovirus was 9.20*10^11^ pfu/ml, and titer of Adeno CMV Null Adenovirus was 3.88*10^11^ pfu/ml.

### Real-time quantitative polymerase chain reaction (RT-qPCR)

Total RNA was extracted from brain tissue using Trizol reagent following the standard protocol (Invitrogen). The extracted RNA was reverse transcribed into cDNA. RT-qPCR was performed according to the published procedure. Primers were designed using Oligo 6.0 software (MBI, Cascade, CO) and were synthesized by Anhui General Bioengineering Co., Ltd. The relative quantitative calculation was 2^−ΔΔCt^. β-actin as internal reference.

The primer sequence was as follows. β-actin: Forward (5’-3’): GCCATGTACGTAGCCATCCA, Reverse (5’-3’): GAACCGCTCATTGCCGATAG, TTCCGTAGCCGGGATTTCGT; PSD-95: Forward (5’-3’):GAGGGGCTTCTACATTAGGGC, Reverse (5’-3’):CTTGACCACTCTCGTCGCTC; Gephyrin: Forward (5’-3’):AATTCTGGTGCAAGCTCGG, Reverse (5’-3’):CATTGAGTAAGTCATCTGGGTTGTC; SYN: Forward (5’-3’):ATTCGAGTACCCCTTCAGGC, Reverse (5’-3’):ACGAGGAGTAGTCCCCAACC, ACATTCGCATCCTGGGTAA. β-actin: Forward (5’-3’):GCCATGTACGTAGCCATCCA, Reverse (5’-3’):GAACCGCTCATTGCCGATAG; CD86: Forward (5’-3’):CCAGGCTCTACGACTTCACA, Reverse (5’-3’):GGTTTCGGGTATCCTTGCTT; CD206: Forward (5’-3’):GGTGCGGTACACTAACTGGG, Reverse (5’-3’):TTCCGTAGCCGGGATTTCGT; TREM2: Forward (5’-3’):TCCTGTTGCTGGTCACAGAG, Reverse (5’-3’):CTCCCATTCTGCTTCCTCAG; DAP12: Forward (5’-3’):CCTGGTGCTTTCTGTTCCTT, Reverse (5’-3’):ACATTCGCATCCTGGGTAA.

### Western blot (WB)

For the extraction of total protein from brain tissue, the BCA method was employed to measure the protein concentration. SDS PAGE gel was prepared and loaded with the protein samples, which were transferred to a PVDF membrane. To prevent non-specific binding, 5% bovine serum albumin was used to seal the membrane. The primary antibodies were added: post synaptic density 95 (PSD-95), synaptophysin (SYN), Gephyrin, CD86, CD206, TREM2, DAP12, p-P38 MAPK, p-ELK-1, ELK-1 and Actin. The samples were incubated overnight at 4℃. secondary antibody was added and incubated, The samples were washed three times with TBST. β-actin served as internal control.

### Statistical analysis

Statistical analysis using SPSS 26.0. Data are expressed as mean ± SD. t test or F test were employed to compare the differences between different groups. When *P* < 0.05, the difference was statistically significant.

## Results

### Prenatally VPA exposure induces ASD behavior in offspring rats

**Fig. 1:**
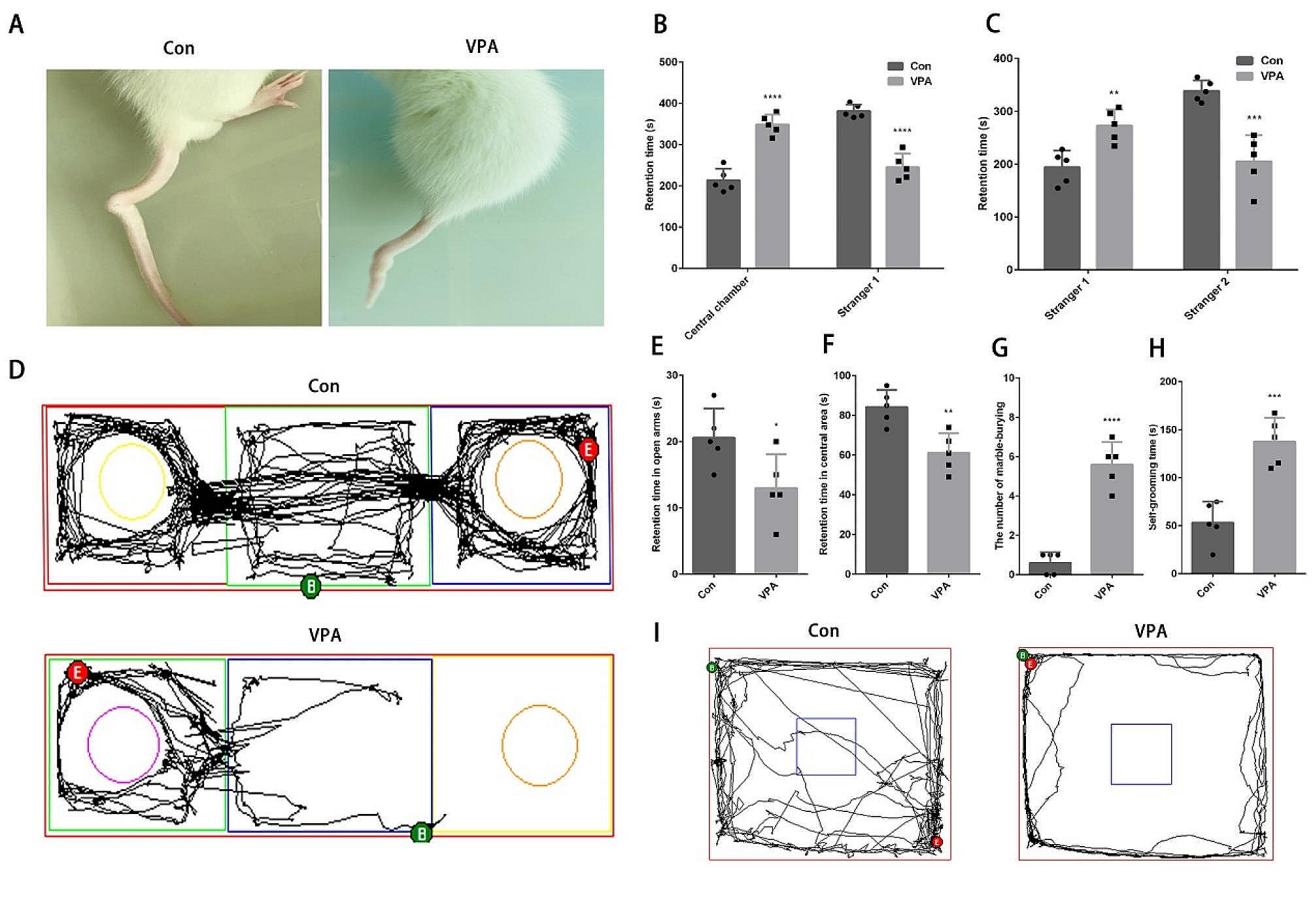
Prenatally VPA exposure induces ASD behavior in offspring rats (**A**) Tail deformity; (**B**) Three-chamber of social ability tracking test (*n* = 5 for each group); (**C**) Three-chamber of social novelty tests (*n* = 5 for each group); (**D**) Trajectory diagram of three-chamber test; (**E**) Elevated plus maze test (*n* = 5 for each group); (**F**) Open field test (*n* = 5 for each group); (**G**) Marble-burying test (*n* = 5 for each group); (**H**) Self-grooming test (*n* = 5 for each group); (**I**) Trajectory diagram of open field test. Data are expressed as mean ± SD. Data were analyzed using the student’s t-test. Compared with the control group, *P < 0.05, ****P < 0.0001

Among the offspring of rats prenatally exposed VPA 600 mg/kg, about 35% of the offspring showed tail deformity, and the tail of the offspring rats was curved and short, which indicated that VPA exposure occurred in uterus (Fig. 1A). Compared with the control group, VPA offspring rats spent longer time in the central chamber and shorter time communicating with stranger 1 (Fig. 1B). The time of olfactory contact communication between VPA offspring rats and stranger 1 was significantly longer than that between VPA offspring rats and stranger 2 (Fig. 1C-D), which indicated that VPA offspring rats showed social ability disorder. Compared with the normal offspring rats, the VPA offspring rats stayed in the open arm for a shorter time, stayed in the central area for a shorter time, and the total number of marbles buried over 2/3 was more (Fig. 1E-G, I), which revealed that the prenatally VPA exposure induced the offspring rats to show a higher level of anxiety. The offspring rats of VPA had a long time of self-grooming, and the offspring rats had repeated rigid behavior (Fig. 1H).

### Prenatally VPA exposure leads to abnormal synaptic development in offspring rats

**Fig. 2:**
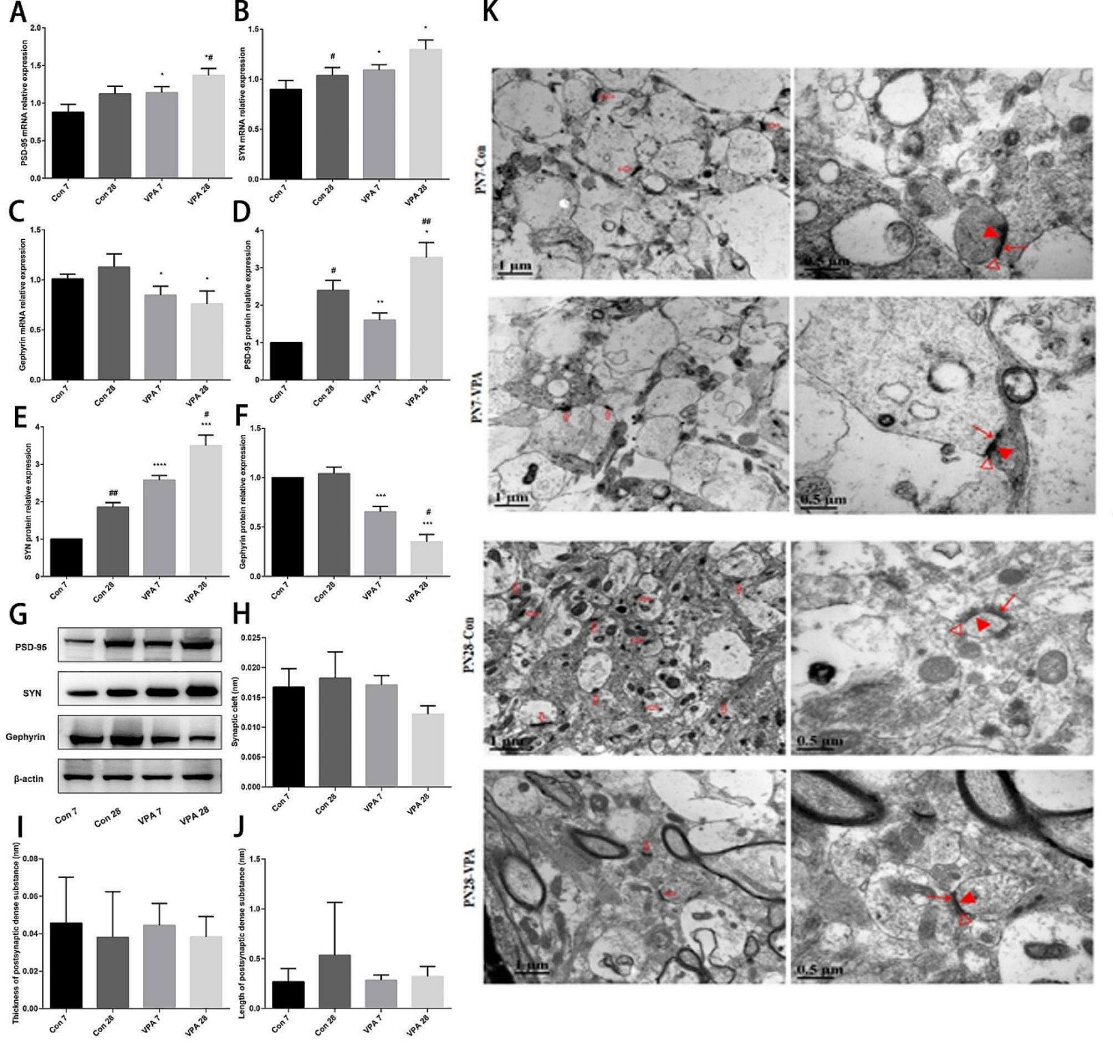
Prenatally VPA exposure leads to abnormal synaptic development in offspring rats (**A**) PSD-95 mRNA expression (*n* = 3 for each group); (**B**) SYN mRNA expression (*n* = 3 for each group); (**C**) Gephyrin mRNA expression (*n* = 3 for each group); (**D**) PSD-95 protein expression (*n* = 3 for each group); (**E**) SYN protein expression (*n* = 3 for each group); (**F**) Gephyrin protein expression (*n* = 3 for each group); (**G**) Protein bands of PSD-95, SYN and Gephyrin (*n* = 3 for each group); (**H**) Synaptic cleft (*n* = 3 for each group); (**I**) Thickness of postsynaptic dense substance (*n* = 3 for each group); (**J**) Length of postsynaptic dense substance (*n* = 3 for each group); (**K**) Synaptic transmission electron micrograph, ⇧synapse, ↑ presynaptic membrane, △ synaptic cleft, ▲ postsynaptic dense substance. Data are expressed as mean ± SD. Data were analyzed using the student’s t-test. Compared with the same age control group, *P < 0.05; Compared with PND 7 in the same group, #*P* < 0.05

RT-qPCR results showed that the expressions of PSD-95 and SYN mRNA in VPA group were higher than those in the control group at PND 7, while Gephyrin mRNA was lower than that in the control group at PND 7. PND 28, the expressions of PSD-95 and SYN mRNA in VPA group were higher than those in the control group, and Gephyrin mRNA was lower than that in the control group, and the expression of PSD-95 mRNA in VPA group was higher than PND 7 (*P* < 0.05) (Fig. 2A-C). WB results showed that PND 7, the expressions of PSD-95 and SYN in VPA group were higher than those in the control group, while Gephyrin protein was lower than that in the control group. PND 28, the expressions of PSD-95 and SYN in VPA group were higher than those in the control group, while Gephyrin protein was lower than that in the control group, and the expression of PSD-95 and SYN protein in VPA group was higher than that in PND 7, and the expression of Gephyrin protein in VPA group was lower than that in PND 7 (*P* < 0.05) (Fig. 2D-F). Typical synaptic structures can be observed under transmission electron microscope. In the control group, the synaptic structure was complete and clear, a small number of vesicles gather in the presynaptic membrane, and the postsynaptic dense substance was long and thick. However, in VPA group, the synaptic structure was vague and incomplete, and the thickness of postsynaptic dense substance was thinner and the length became shorter. But there was no significant difference in synaptic cleft, thickness and length of postsynaptic dense substance between the control group and VPA group (*P* > 0.05) (Fig. 2G-J).

### Prenatally VPA exposure changes the phenotype of microglia in offspring rats

**Fig. 3:**
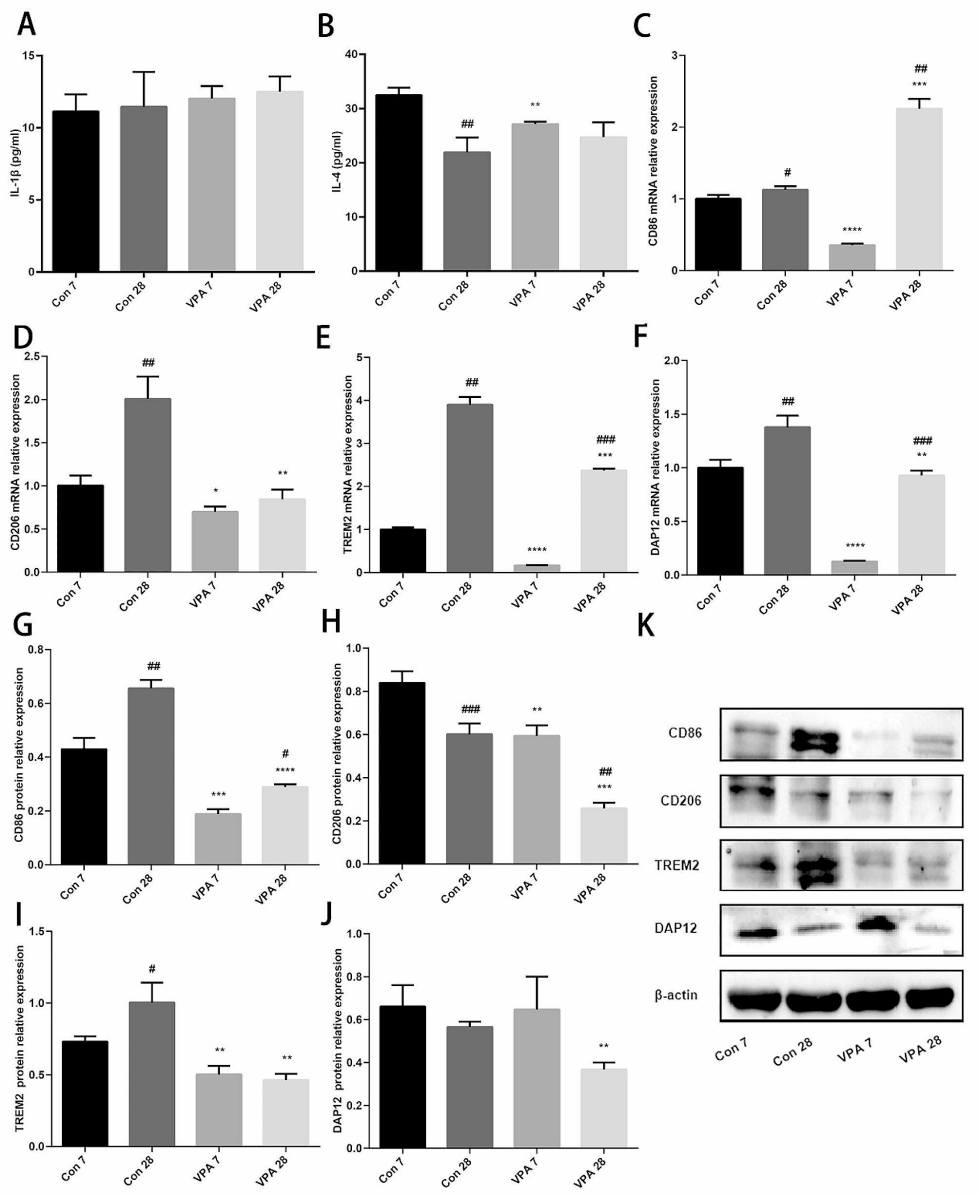
Prenatally VPA exposure changes the phenotype of microglia in offspring rats (**A**) IL-1β (*n* = 3 for each group); (**B**) IL-4 (*n* = 3 for each group); (**C**) Expression of CD86 mRNA (*n* = 3 for each group); (**D**) Expression of CD206 mRNA (*n* = 3 for each group); (**E**) Expression of TREM2 mRNA (*n* = 3 for each group); (**F**) Expression of DAP12 mRNA (*n* = 3 for each group); (**G**) Expression of CD86 protein (*n* = 3 for each group); (**H**) Expression of CD206 protein (*n* = 3 for each group); (**I**) Expression of TREM2 protein (*n* = 3 for each group); (**J**) Expression of DAP12 protein (*n* = 3 for each group); (**K**) Protein bands of CD86, CD206, TREM2 and DAP12. Data are expressed as mean ± SD. Data were analyzed using the student’s t-test. Compared with the same age control group, *P < 0.05, **P < 0.01, ***P < 0.001, ****P < 0.0001; Compared with PND 7 in the same group, #*P* < 0.05, ##*P* < 0.01, ###*P* < 0.001, ####*P* < 0.0001

**Fig. 4:**
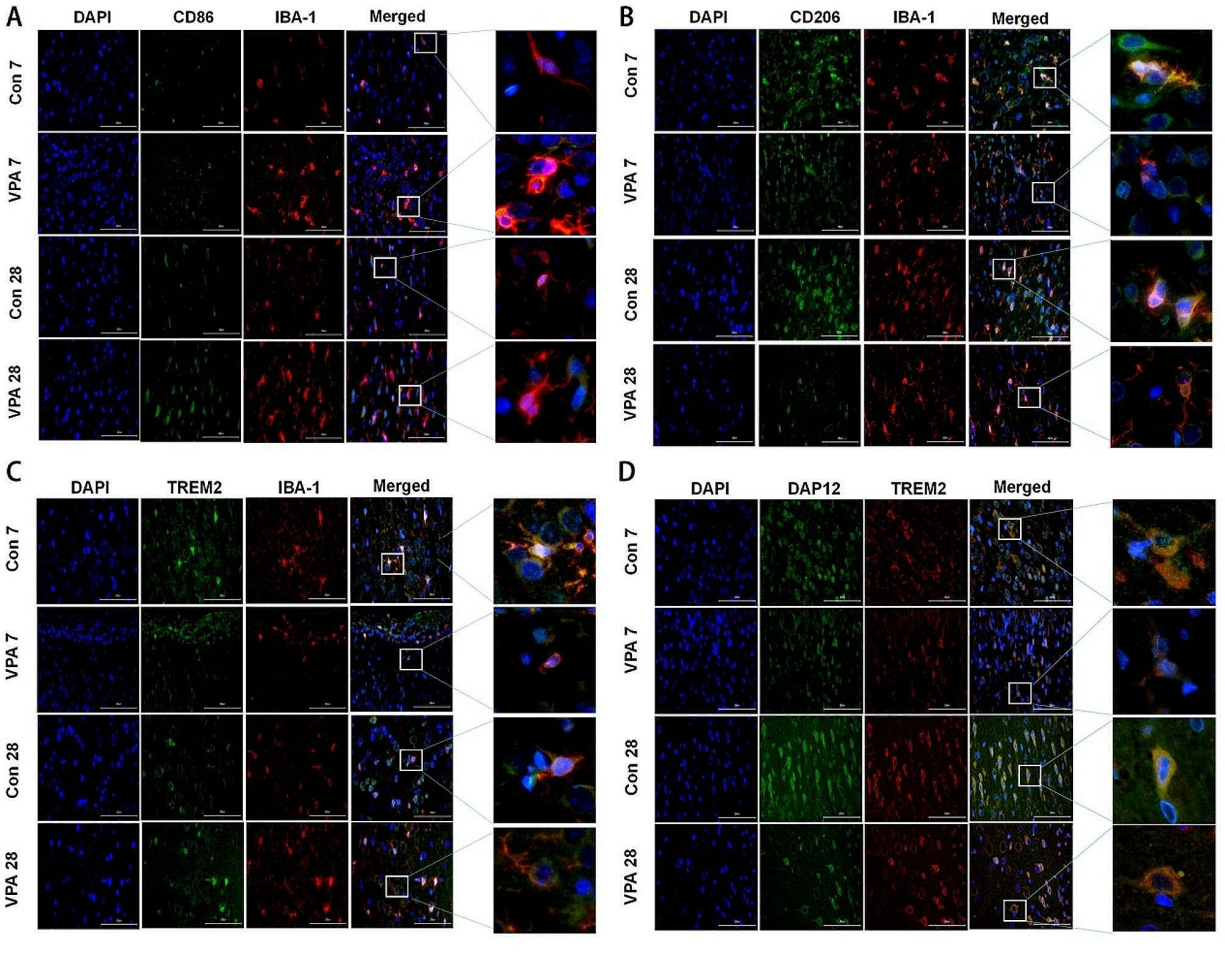
Immunofluorescence staining diagram of microglia phenotype in PFC area (**A**) CD86; (**B**) CD206; (**C**) TREM2; (**D**) DAP12; scale bars = 100 μm

Firstly, the author used ELISA to detect the levels of IL-1β and IL-4 in PFC brain tissue, and it was found that there was no difference in IL-1β levels between the two groups at different time points (*P* > 0.05). At PND 7, the level of IL-4 in VPA group was significantly lower than that in control group (*P* < 0.05) (Fig. 3A-B). Then, RT-qPCR results showed that at PND 7, the expressions of CD86, CD206, TREM2 and DAP12 mRNA in VPA group were lower than those in the control group, at PND 28, the expression of CD86 mRNA in VPA group was higher than that in control group, the expressions of CD206, TREM2 and DAP12 mRNA were lower than that in control group (*P* < 0.05) (Fig. 3C-F). In addition, the author examined the protein expression of microglial cell markers and found that whether in PND 7 or PND 28, the protein contents of CD86, CD206 and TREM2 in VPA group were significantly lower than those in control group (*P* < 0.05); at PND 28, the protein content of CD86 in VPA group was higher than that of PND 7, and the protein content of CD206 was lower than that of PND 7; at PND 28, the content of DAP12 protein in VPA group was significantly lower than that in control group (*P* < 0.05) (Fig. 3G-K). Immunofluorescence detection showed that at PND 7, compared with the control group, the relative fluorescence intensity of the co-location of TREM2/IBA-1 fluorescent protein expression in VPA group was significantly weakened, and the expression of TREM2/DAP12 was significantly down-regulated in both PND 7 and PND 28 (*P* < 0.05). CD86 increased in VPA rats, and CD206 decreased in VPA rats, but there was no significant difference between the two groups (*P* > 0.05) (Fig. 4A-D).

### TREM2 regulates microglia function through P38 MAPK pathway

**Fig. 5:**
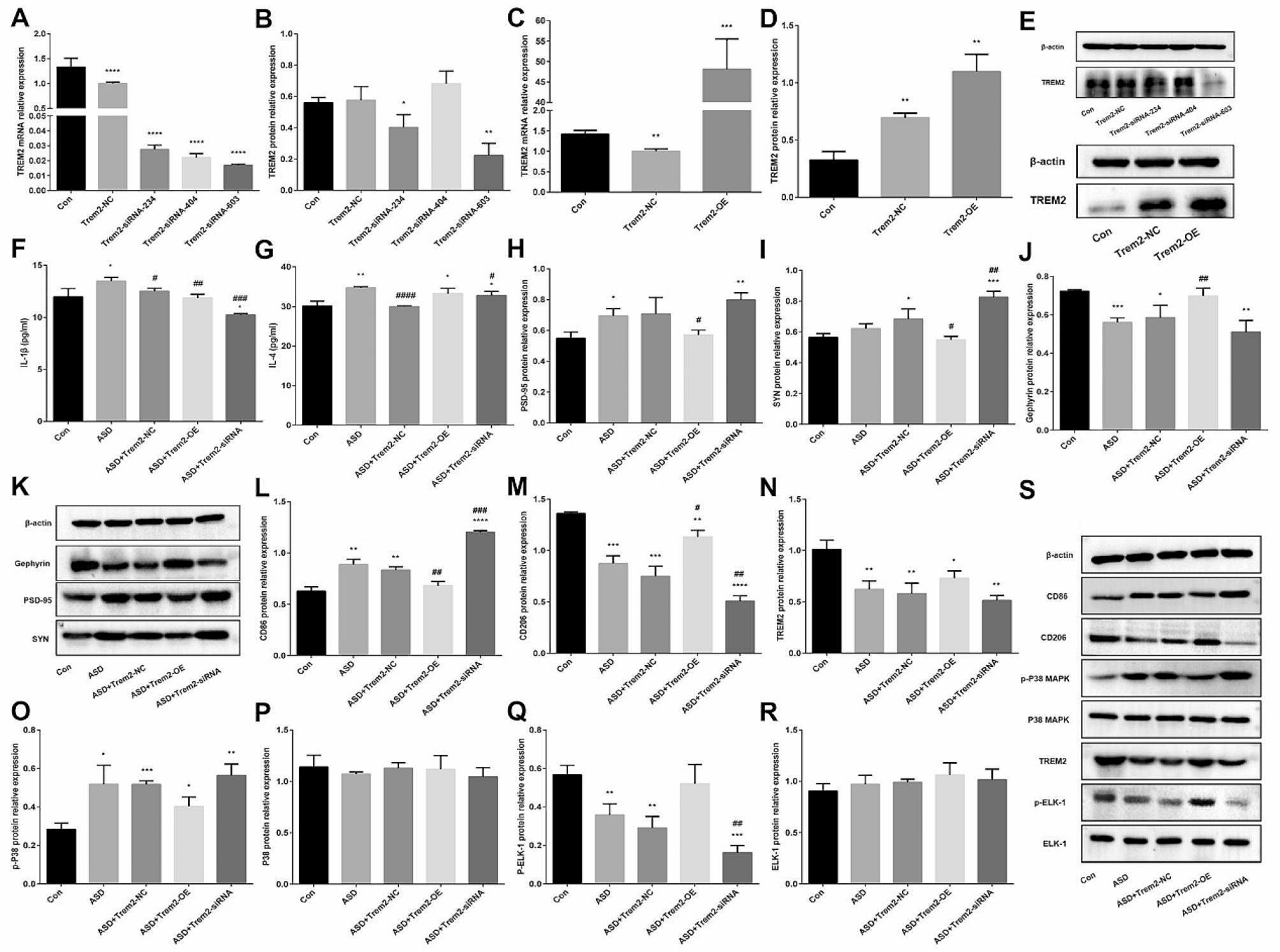
TREM2 regulates microglia function through P38 MAPK pathway (**A**) After TREM2 interference, TREM2 mRNA expression (*n* = 3 for each group); (**B**) After TREM2 interference, TREM2 protein expression (*n* = 3 for each group); (**C**) After TREM2 overexpression, TREM2 mRNA expression (*n* = 3 for each group); (**D**) After TREM2 overexpression, TREM2 protein expression (*n* = 3 for each group); (**E**) Protein bands of TREM2 interference and overexpression; (**F**) IL-1β (*n* = 3 for each group); (**G**) IL-4 (*n* = 3 for each group); (**H**) PSD-95 protein expression (*n* = 3 for each group); (**I**) SYN protein expression (*n* = 3 for each group); (**J**) Gephyrin protein expression (*n* = 3 for each group); (**K**) Protein bands of PSD-95, SYN and Gephyrin; (**L**) CD86 protein expression (*n* = 3 for each group); (**M**) CD206 protein expression (*n* = 3 for each group); (**N**) TREM2 protein expression (*n* = 3 for each group); (**O**) p-P38 MAPK protein expression (*n* = 3 for each group); (**P**) P38 MAPK protein expression (*n* = 3 for each group); (**Q**) p-ELK-1 protein expression (*n* = 3 for each group); (**R**) ELK-1 protein expression (*n* = 3 for each group); (**S**) Protein bands of CD86, CD206, TREM2, p-P38 MAPK, P38 MAPK, p-ELK-1 and ELK-1. Data are expressed as mean ± SD. Data were analyzed using the F-test and t-test. Compared with the control group, *P < 0.05, **P < 0.01, ***P < 0.001, ****P < 0.0001; Compared with the ASD group, #*P* < 0.05, ##*P* < 0.01, ###*P* < 0.001, ####*P* < 0.0001

In order to further prove that TREM2 is involved in the pathogenesis of ASD, the author constructed a cell model of TREM2 interference and overexpression by lentivirus and adenovirus. RT-qCPR and WB experiments verified that TREM2 was effectively inhibited or overexpressed, siRNA-603 was selected for follow-up experiments (Fig. 5A-E). Compared with the control microglia, the levels of IL-1β and IL-4 in ASD microglia increased significantly. The level of IL-1β in ASD + Trem2-OE group was lower than that in ASD group, and the level of IL-4 did not change significantly. Compared with ASD group, ASD + Trem2-NC group and ASD + Trem2-siRNA group have lower levels of IL-4 (*P* < 0.05) (Fig. 5F-G). WB results showed that the expression of PSD-95 and SYN protein in ASD + Trem2-siRNA group were higher than those in control group, and the expression of Gephyrin protein was lower than that in control group. The expressions of PSD-95 and SYN protein in ASD + Trem2-OE group were lower than those in ASD group, and the expression of Gephyrin protein was higher than that in ASD group (*P* < 0.05) (Fig. 5H-K). Compared with the control group, the content of CD86 protein in ASD group, ASD + Trem2-NC group and ASD + Trem2-siRNA group increased significantly, while the content of CD86 protein in ASD + Trem2-OE group was significantly lower than that in ASD group. The expression of CD206 and TREM2 protein in ASD + Trem2-siRNA group was lower than that in control group, and the expression of CD206 protein was lower than that in ASD group, and the content of CD206 protein in ASD + Trem2-OE group was significantly higher than that in ASD group (*P* < 0.05) (Fig. 5L-N, S). In addition, the author also found that compared with the control group, the content of p-P38 MAPK protein in ASD group increased and the content of p-ELK-1 protein decreased. After interfering with the expression of TREM2, the content of p-P38 MAPK protein increased and the content of p-ELK-1 protein decreased (*P* < 0.05) (Fig. 5O-R, S).

## Discussion

Patients with ASD often show difficulties in communicating with others, backward language skills, frequent stereotyped movements, and refusal to accept changes. Their pathogenesis is affected by the intrauterine environment during pregnancy, and the disease usually accompanies them for life. As a histone deacetylase inhibitor, VPA has a good regulatory effect on GABA and has been widely used in the treatment of epilepsy and bipolar disorder [13]. However, taking VPA during pregnancy has been proved to be harmful, which will affect the neural development of the fetus, lead to obvious risk of fetal malformation, and cause birth defects such as cognitive impairment and ASD [14]. In this study, the rat model was established by intraperitoneal injection of VPA, and found that among the offspring of rats prenatally exposed VPA, about 35% of the offspring showed tail deformity, which indicated that VPA exposure occurred in uterus and could cause developmental defects of offspring rats through placenta. Behavioral experiments observed that prenatally VPA exposure induces offspring rats to show more obvious typical ASD core symptoms such as social dysfunction, anxiety-like behavior and repetitive rigid behavior.

One potential etiology of ASD is a disorder in nervous system development, manifesting as alterations in neuronal count, aberrant morphology, and compromised functional integration. It has been found that VPA can interfere with the differentiation of GABAergic intermediate neurons, increase the number of excitatory synapses, and decrease the number of inhibitory synapses, which leads to the continuous change of synaptic protein expression and ultimately affects the functional activities of synapses [15–16]. PSD-95, SYN and Gephyrin are all important indexes related to synaptic function. PSD-95 is a scaffold protein located in the dense area after excitatory synapse, which is involved in regulating the formation and function of excitatory synapse and can reflect the signal integration ability of synapse, and is highly related to the density of dendritic spines and synaptic plasticity. SYN mainly exists in the presynaptic vesicle membrane of neurons, reflecting the integrity and functional state of synaptic structure and the density and distribution of synapses. Gephyrin is a scaffold protein in the inhibitory postsynaptic dense area, which plays an important role in the regulation of inhibitory synaptic formation, plasticity and nerve transmission. Some scholars believe that the excitatory/inhibitory synaptic balance is very important for normal cerebral cortex function, and there is an excitatory/inhibitory synaptic imbalance in ASD patients, which may be related to the enhancement of glutamate synaptic transmission in the dorsal raphe nucleus [17]. The author’s research results showed that prenatally VPA exposure led to abnormal expression of synapse-related proteins in PFC tissue of offspring rats, increased excitatory synapses and decreased inhibitory synapses, which led to abnormal synaptic development. In addition, synaptic structural indicators such as synaptic cleft, thickness of postsynaptic dense substance and length of postsynaptic dense substance can effectively reflect the information transmission efficiency of synapses. Neurotransmitters diffuse in the synaptic cleft and transmit signals to another neuron. As the central link of synaptic level signal integration, postsynaptic dense matter can change the downstream effect of nerve signal transmission, and its thickness and length can reflect the state of neuronal functional activity to some extent [18]. In this study, it was found that prenatally VPA exposure induces certain irregularities in synaptic development.

Microglia are resident immune cells in the central nervous system, which participate in the growth, pruning, survival and synaptic formation of neurons and maintain the steady state of the nervous system [19]. The function of microglia can be divided into neurotoxicity or neuroprotection. Microglia with pro-inflammatory phenotype can promote the secretion of inflammatory cytokines such as IL-1β and increase the expression of markers such as CD86, thus triggering an immune inflammatory cascade reaction, while microglia with anti-inflammatory phenotype can inhibit the occurrence of inflammatory reaction, secrete IL-4 and nerve growth factor, and generate markers such as CD206. It has been reported that the increased levels of IL-1β and IL-4 are related to the development of neonatal ASD, and the abnormal levels of IL-1β and IL-4 may indicate the genetic differences and environmental exposure related to ASD [20]. This study revealed that VPA could affect the balance of pro-inflammatory and anti-inflammatory reactions. In addition, TREM2 expression in microglia has been found to have beneficial effects on tissue repair, apoptotic cell phagocytosis, and anti-inflammatory responses [21]. TREM2 lacks a signal transduction or transport motif in its short cytoplasmic tail. However, DAP12, serving as a linker protein for TREM2, possesses an immune receptor tyrosine activation motif in its cytoplasm. Upon binding to DAP12, TREM2 initiates downstream signaling pathways, including PI3K and ERK, thereby regulating microglial function [22]. This study found that prenatally VPA exposure changes the phenotype of microglia in offspring rats, promotes the polarization of microglia to pro-inflammatory type, and increases the inflammatory response.

In order to further prove that TREM2 is involved in the pathogenesis of ASD, primary microglia and primary neuron cells were successfully cultured and identified in this study, and TREM2 in microglia was interfered and overexpressed by lentivirus and adenovirus. In vitro experiments of this study showed that the overexpression of TREM2 could alleviate some inflammatory reactions and maintain the elasticity of pro-inflammatory and anti-inflammatory functions. Wu et al. found that the overexpression of TREM2 can not only increase the density of dendritic spines, promote dendritic complexity, but also inhibit neuroinflammation and microglia activation [23], which is broadly consistent with the results of this study. After interfering with TREM2, the synaptic related proteins of ASD microglia are out of balance, which has adverse effects on synaptic development; After overexpression of TREM2, the expression of Gephyrin protein was reversed, indicating that TREM2 can increase the expression of Gephyrin and improve the synaptic development of neurons, which may be one of the reasons why TREM2 improves the behavior of ASD rats induced by VPA.

The vitro experimental results of this study also showed that TREM2 played a role in the conversion of microglia into the anti-inflammatory phenotype, and the overexpression of TREM2 exhibited the potential to restore the polarization of microglia associated with ASD. The results of immunofluorescence also confirmed this conclusion. Liu et al. reported that the knockdown of TREM2 weakened the response of microglia to IL-4, and TREM2 participated in the pathological process of Alzheimer’s disease by promoting the phenotype polarization of microglia to anti-inflammatory phenotype [24]. Ulland et al. found that in the mouse model of TREM2 knockout, microglia will produce a large number of autophagic vesicles, which are characterized by neuronal malnutrition and energy metabolism disorder. TREM2 can maintain microglia survival by regulating cell energy and biosynthesis metabolism, thus improving microglia function [25]. Zhai et al. used lipopolysaccharide to induce the pro-inflammatory phenotype of microglia, and found that the expression of TREM2 decreased, while TREM2 was highly expressed in cells with anti-inflammatory phenotype, TREM2 played a neuroprotective role by regulating the phenotypic transformation of microglia and the release of inflammatory cytokines [26].

Furthermore, P38 MAPK is the second MAPK family activated by stress in mammals, which plays a crucial role in inflammation, neuronal apoptosis, ischemia-reperfusion injury and synaptic plasticity [27]. ELK-1 transcription factor is a specific downstream target of P38 MAPK pathway, MAPK pathway regulates the activity of ELK-1 through phosphorylation and ubiquitination [28]. By exposing mouse BV2 microglia to hypoxia and high glucose, Zhang et al. thought that knocking out TREM2 could enhance the activation of p38 MAPK signal and aggravate the neuroinflammatory reaction mediated by P38 MAPK signal [29]. Ruganzu et al. reported that TREM2 was involved in the cognitive decline of Alzheimer’s disease by influencing microglia proliferation and loss of neurons and synapse-related proteins, and interfering with neuroinflammatory reaction through MAPK signaling pathway mediated by TLR4 [30]. Currently, the regulatory function of TREM2 in various diseases via P38 MAPK pathway has been discussed, but its involvement in ASD remains unexplored. The author’s research results revealed that TREM2 improves microglial function in ASD by regulating the P38 MAPK signaling pathway.

To sum up, the protective influence of TREM2 on the VPA-induced ASD model is attributed to its inhibition of the P38 MAPK pathway, this protective effect may be achieved by promoting the polarization of microglia to anti-inflammatory phenotype and improving the neuronal synaptic development. The limitation of this study is that only in vitro siRNA and overexpression methods are used to verify the function of TREM2, and the influence of other factors on the results is not considered. This study needs to use TREM2 KO mice to study the biological consequences of TREM2 dysfunction in the future, and it is still necessary to explore the changes of TREM2 in the whole brain region in the future, and exclude interference caused by maternal factors such as maternal autoimmune, inflammation, and chronic stress.

## Data Availability

The data that support the findings of this study are available from the corresponding author Xiaoping Zhu, upon reasonable request.
